# GSA Central—A web platform to perform, learn, and discuss gene set analysis

**DOI:** 10.3389/fmed.2022.965908

**Published:** 2022-08-11

**Authors:** Xiaowei Huang, Xuanyi Lu, Chengshu Xie, Shaurya Jauhari, Zihong Xie, Songqing Mei, Antonio Mora

**Affiliations:** ^1^Joint School of Life Sciences, Guangzhou Medical University and Guangzhou Institutes of Biomedicine and Health (Chinese Academy of Sciences), Guangzhou, China; ^2^School of Biomedical Engineering, Guangzhou Medical University, Guangzhou, China

**Keywords:** web platform, gene set analysis, pathway analysis, database, galaxy, benchmarking, education

## Abstract

Gene Set Analysis (GSA) is one of the most commonly used strategies to analyze *omics* data. Hundreds of GSA-related papers have been published, giving birth to a GSA field in Bioinformatics studies. However, as the field grows, it is becoming more difficult to obtain a clear view of all available methods, resources, and their quality. In this paper, we introduce a web platform called “GSA Central” which, as its name indicates, acts as a focal point to centralize GSA information and tools useful to beginners, average users, and experts in the GSA field. “GSA Central” contains five different resources: A Galaxy instance containing GSA tools (“Galaxy-GSA”), a portal to educational material (“GSA Classroom”), a comprehensive database of articles (“GSARefDB”), a set of benchmarking tools (“GSA BenchmarKING”), and a blog (“GSA Blog”). We expect that “GSA Central” will become a useful resource for users looking for introductory learning, state-of-the-art updates, method/tool selection guidelines and insights, tool usage, tool integration under a Galaxy environment, tool design, and tool validation/benchmarking. Moreover, we expect this kind of platform to become an example of a “thematic platform” containing all the resources that people in the field might need, an approach that could be extended to other bioinformatics topics or scientific fields.

## Introduction

“Gene Set Analysis” (GSA) is an annotation-based approach for *omics* data analysis. It has been defined as the statistical comparison of a query gene set to a database of annotated gene sets to transform gene-level experimental results into gene-set-level experimental results. In other words, a statistical method to interpret a query gene set in terms of biological pathways or functionally related gene sets from a reference database (1). If the query set is made of genes differentially expressed between two experimental conditions, a GSA result can be understood as the gene sets, ontology terms, or pathways significantly enriched between those experimental conditions (1).

GSA has become one of the standard analyses of current *omics* data analysis workflows. Therefore, numerous independent tools have been created to perform GSA on different types of datasets or from different programming environments, such as GSEA (2), DAVID (3), Enrichr (4), clusterProfiler (5), GOseq (6), and ClueGO (7). Multiple reviews have been written and multiple courses have been offered, and, consequently, a GSA sub-field with its own jargon, methods, tools, and opposing schools has appeared. In this context, our goal is to create a web platform that can serve as a focal point for GSA practitioners, both novice and experts. In this platform, biomedical researchers can use simplified versions of the existing tools, find information about all existing methods and reviews, discuss new developments, follow online lessons, and more.

**FIGURE 1:**
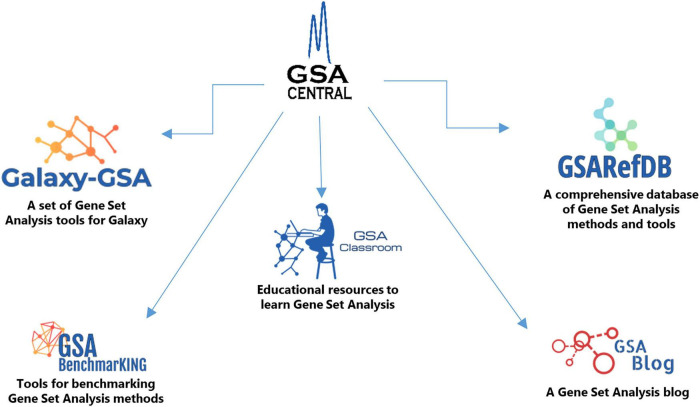
GSA Central architecture.

Here we introduce “GSA Central,” a web platform to perform, learn, and discuss GSA, which is divided into five sections (Figure 1): (i) “Galaxy-GSA” (a collection of a variety of GSA tools inside a Galaxy environment), (ii) “GSA Classroom” (a database of online GSA courses and videos), (iii) “GSARefDB” (a comprehensive database of all published GSA papers), (iv) “GSA BenchmarKING” (a repository of tools to benchmark GSA methods and software), and (v) “GSA Blog” (a place to discuss novel GSA-related topics).

## Materials and methods

### GSA Central

GSA Central’s website was built as a GitHub website^1^ using HTML, CSS, and javascript. The code is open source and can be reviewed or cloned from https://github.com/gsa-central/gsa-central.github.io.

### Galaxy-GSA

Galaxy is one of the most popular multi-purpose bioinformatics platforms. It was originally built as a platform for researchers without programming experience and started as a public server with an emphasis on simplicity.^2^ The original Galaxy server offered data search and manipulation options, multiple bioinformatics tools, and the possibility of easily combining different analyses thanks to an element called the “History” which allowed the storage of every intermediate result (8). Soon after, it was clear that Galaxy offered more than simplicity, and quickly became one of the best platforms to guarantee the transparency and reproducibility of full computational analyses for biologists and bioinformaticians (9). More recently, Galaxy has evolved into an open environment that facilitates the development of independent and specialized Galaxy instances and servers, as well as the wrapping of multiple bioinformatics packages or libraries inside the Galaxy framework to take advantage of its simplicity, transparency, and reproducibility. Due to that, a vast ecosystem of Galaxy servers^3^ and individual tools^4^ has been built, which nowadays includes Galaxy tools for the most common tasks found in Bioinformatics.

Several GSA tools have been added to the Galaxy public server, including “g:Profiler” (10), “DAVID” (3), “goseq” (6), “EGSEA” (11), “fgsea” (12), “GOEnrichment” (13), and “KOBAS” (14). Using the galaxy toolshed, it is possible to find other popular GSA tools authored by the community, such as “clusterProfiler” (5) and “TopGO” (15). However, other kinds of experimental designs may require more sophisticated GSA tools. For example, an experiment with thousands of patients and only one sample per patient would be better suited for using “single-sample” GSA methods; an experiment generating multi-omics datasets would better use “integrative” GSA methods; *omics* data different to mRNA, such as ncRNA, ChIP-seq, or methylation data, would need an additional mapping procedure before the specific GSA method is applied; and, following the same reasoning, we can think of multiple scenarios that justify using specialized types of GSA methods. Therefore, we created a set of galaxy tools that we called “Galaxy-GSA” to fulfill the need for such methods inside the Galaxy environment. Galaxy-GSA wraps multiple R packages into Galaxy and gives them the chance to communicate with each other and with all other Galaxy tools. We have made such tools individually available in the galaxy toolshed and collectively available as a virtual machine, a Docker image, and a web platform. Also, we have distributed a few Galaxy workflows for specific scenarios and practical tasks. All of this will allow the user to leverage the results of both general and specialized GSA methods inside Galaxy’s environment.

All Galaxy-GSA tools are initially written in R and integrated into Galaxy using XML and tools such as Planemo.^5^ Full tutorials are included on our website with detailed explanations on how to use Galaxy-GSA,^6^ how we built our Galaxy instance,^7^ and how we created the new Galaxy tools.^8^

### GSA Classroom

For beginners, educational material for learning GSA methods and tools might be the most important resource.

There are multiple educational slides and videos scattered all over the Internet. We have collected information and links to such resources in a single place, including courses (slides), courses (animations), and online videos. Regarding online videos, we have added a search engine to facilitate GSA video search according to several descriptors, including: title, author, keywords, language (English or Chinese), video platform, and year, besides the links to such resources.

GSA Classroom can be accessed at: https://gsa-central.github.io/education.html. The animated GSA lessons were created using Vyond^9^ and Adobe After Effects.^10^ The video search engine was built using R 4.0.0 and its “shiny” package. Its code is open source and can be accessed at https://github.com/gsa-central/gsaclassroom. The shiny app reads the information from an excel table located at its “data” folder^11^; therefore, the app can be easily updated by updating such table.

### GSARefDB

Some GSA reviews have previously tried to reference all existing GSA methods and tools; however, previous to our work, the most comprehensive report included merely 68 GSA tools (16). In 2019, we introduced the “Gene Set Analysis Reference Database” (GSARefDB), the most comprehensive compilation of available methods, tools, and reviews for GSA. GSARefDB is not only a list of papers but also includes valuable meta-data, such as references (author, title, year), classification (types of GSA methods), popularity (citation counts), method details, and other descriptors. Our first version included 445 papers [version used in Mora (17)], while our second version contained 503 papers [version used in Xie et al.(1)]. Our most recent version contains 641 papers, which makes GSARefDB the largest database of GSA methods and tools. In addition, its associated meta-data (such as method classification and popularity) makes it one of a kind.

GSARefDB was initially built as an excel spreadsheet. Citation information was extracted from Google scholar, while all other information was manually extracted from the papers. The spreadsheet versions of the database can be downloaded at https://github.com/gsa-central/gsarefdb/tree/master/archive/. We also created a “GSARefDB” app, which can be found at https://gsa-central.github.io/gsarefdb.html. The app was built using R version 3.6.3 and “shiny” version 1.4.0.2. Its code is open source and can be accessed at https://github.com/gsa-central/gsarefdb.

### GSA BenchmarKING

Computational method or tool selection is fundamental to the process of generating high-quality scientific results, and the two most common selection strategies are (i) following the method’s popularity or (ii) following objective performance tests. GSARefDB is the best existing resource to find out the popularity of GSA tools. However, popular methods are not necessarily the best and, therefore, we should always consider the recommendations of performance studies, such as benchmark and simulation studies (1). That is not an easy task because the existing benchmarks are very few and only evaluate a few methods at the time [see Table 1 of Xie et al. (1)], while performing new benchmark studies is a labor-intensive task. Because of that, we have introduced some benchmarking guidelines, together with “GSA BenchmarKING,” which is a repository of tools to perform an easy benchmark of GSA tools.

The “GSA-BenchmarKING” repository^12^ stores tools to measure GSA method performance which are expected to have the following attributes: (i) Be open software; (ii) Have a clear reason for selecting the group of GSA methods under comparison; for example, because all of them belong to the same type of methods; (iii) Include both a gold standard dataset and options to upload user-selected gold standard datasets (an example of this is showed in the Results section, GSA BenchmarKING sub-section); (iv) Include either a list of “target pathways” linked to the gold standard dataset or “disease relevance scores” per pathway for the diseases related to the gold standard [this is explained and discussed in Xie et al. (1)]; (v) Give the user the option of selecting different benchmarking metrics (such as precision, sensitivity, prioritization, and specificity); (vi) Options for selecting ensemble results; (vii) Flexibility for easily adding new GSA methods to the code in the future.

The current tools in GSA BenchmarKING include jupyter notebooks and shiny apps for benchmarking both “single-sample GSA” tools and “genomic-range GSA” tools. The “ss-shiny” app was built using R 3.6.2, while the “gr-shiny” app was built using R 4.0.0. Their code is open source and can be accessed at https://github.com/mora-lab/ss-shiny and https://github.com/mora-lab/gr-shiny, respectively. The performance metrics (precision, sensitivity, and specificity) are defined as below:


Precision=TP/TP+FP

Sensitivity=TP/TP+FN

Specificity=TN/TN+FP

where TP = True Positives, FP = False Positives, TN = True Negatives, and FN = False Negatives.

Our benchmark studies always include an ensemble of all methods under consideration, which is built by combining their individual *p*-values. The combination of *p*-values is performed through the “metap” R package, which allows the user to choose between Fisher’s method (sum of log), Stouffer’s method (sum of z), or a simple average of *p*-values.

### GSA Blog

We have created the GSA Blog as a space where both beginners and experts can find discussions on topics of interest in the field. The GSA Blog was also built as a GitHub website,^13^ using HTML, CSS, and javascript. The code is open source and can be reviewed or cloned from https://github.com/gsa-blog/gsa-blog.github.io.

## Results

### Galaxy-GSA

Galaxy-GSA is a collection of Gene Set Analysis tools for different types of Bioinformatics projects inside a Galaxy environment. It is built as a toolbox that contains original tools, wrappers for existing R packages, and workflows with various goals. Users are offered several options for functional interpretation of *omics* data to choose one depending on the goals of their study. For example, having mRNA data, transcription factor data, or multiple *omics* datasets; having several replicates of a given cell line or just one sample for each of many patients; focusing on pathway structure or ontology term annotation; and so on. Galaxy-GSA includes (i) popular general-purpose GSA tools, (ii) specialized tools built for specific experimental designs, (iii) specialized tools for datasets different from mRNA, (iv) GSA-related auxiliary tools, and (v) Workflows for specific types of Bioinformatics projects.

The current tools include:

(1) Gene set uploader: Tool to select a collection of gene sets or pathways from either MSigDB or KEGG and use them as reference gene sets for other gene set analysis tools.
(2) ReactomePA: Over-representation analysis tool that finds over-representation of Reactome pathways in a list of Entrez gene IDs through the hypergeometric model (18).
(3) SPIA: Pathway-Topology tool that considers the topology of the pathways by combining an over-representation score with a perturbation factor (which, in turn, combines the fold change of a gene with the fold change of the genes upstream) (19).
(4) GSVA: Tool containing four different single-sample (or sample-specific) methods: PLAGE, ZSCORE, SSGSEA, and GSVA. Such methods compute single-sample-specific enrichment statistics and do not generate a single pathway ranking but a sample-pathway matrix instead (20).
(5) ChIPEnrich: Tool containing four different GSA methods for genomic regions (i.e., peaks coming from ChIP-seq and similar technologies). ChIPEnrich and PolyEnrich can be applied to narrow peaks such as transcription factor binding sites, while BroadEnrich has been designed to work with broad peaks such as histone marks (21).
(6) methylGSA: Tool that performs GSA for DNA methylation data using logistic regression (22).
(7) mogsa: Tool that performs GSA on multiple *omics* data by integrative clustering (23).
(8) GSAR: Tool that includes a series of methods that compare hypotheses different to the equality of means of a gene set between two conditions. For example, WW is a test of differential distribution; the aggregated *F*-test is a test of differential variance; and GSNCA is a test of differential co-expression that measures the change in the net correlation structure (24).

Each Galaxy-GSA tool gives visibility to all the software options, parameters, and help from the original packages. The user can leverage Galaxy’s infrastructure to upload data, format data, choose parameters and options through a simple interface, and send results to the history to allow comparisons between different runs or link results to other bioinformatics tools. The current Galaxy-GSA server version contains one original tool for downloading gene sets and seven wrappers for R packages. It has been organized into different GSA categories, and those categories also include other GSA-related Galaxy tools not developed for us, such as g:Profiler, DAVID, and others.

For experienced Galaxy users, we have built three solutions:

(i) Individual Galaxy-GSA tools can be downloaded from the “Galaxy toolshed”,^14^
(ii) A Galaxy-GSA Docker image can be downloaded from “Docker hub”,^15^
(iii) A virtual machine image can be downloaded from Zenodo.^16^

For new Galaxy users, we have provided instructions on how to install Galaxy, and we have built a Galaxy-GSA platform on our website.^17^ The website is ideal for beginners and quick testing, as it does not require any installation whatsoever; however, it does not allow saving your histories or installing new tools.

**FIGURE 2:**
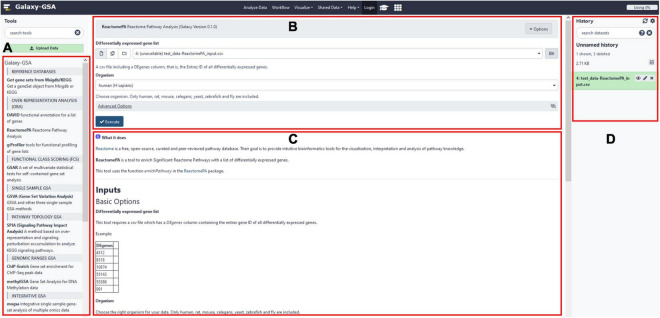
Galaxy-GSA screenshot. **(A)** All installed Galaxy tools (including all Galaxy-GSA tools). **(B)** Input boxes for the currently open tool (here, ReactomePA). **(C)** Help for the currently open tool. **(D)** History (including intermediate and final results).

Besides that, Galaxy-GSA has also been included in the Galaxy Platform Directory (see text footnote 3) as one of the 125 Galaxy platforms currently available. You can find Galaxy-GSA under “Public Servers,” “Containers,” and “VMs.” A list of Galaxy-GSA implementations and individual tools is shown in Table 1. Figure 2 shows screenshots of Galaxy-GSA.

**TABLE 1:** A list of current servers and individual tools for Galaxy-GSA.

	Tool name	Tool type	Location
1	Galaxy-GSA	WEB	https://gsa-central.github.io/galaxy.html
2	Galaxy-GSA	Docker	https://gsa-central.github.io/galaxy.html
3	Galaxy-GSA	Virtual machine	https://gsa-central.github.io/galaxy.html
4	Gene set uploader	Data uploader	Toolshed
5	ReactomePA	ORA	Toolshed
6	SPIA	PT	Toolshed
7	PLAGE	SS	Toolshed
8	ZSCORE	SS	Toolshed
9	SSGSEA	SS	Toolshed
10	GSVA	SS	Toolshed
11	ChIPEnrich	GR	Toolshed
12	PolyEnrich	GR	Toolshed
13	BroadEnrich	GR	Toolshed
14	methylGSA	GR	Toolshed
15	Mogsa	INTEG	Toolshed
16	WW	FCS	Toolshed
17	KS	FCS	Toolshed
18	Agg-F	FCS	Toolshed
19	GSNCA	FCS	Toolshed

WEB, Web platform; Docker, Docker image; VM, Virtual Machine; ORA, Over-representation analysis; FCS, Functional-class scoring; PT, Pathway topology-based; SS, Single-sample; GR, Genomic Region; INTEG, Integra-tive; Toolshed, Galaxy toolshed (https://galaxyproject.org/toolshed/ or https://toolshed.g2.bx.psu.edu/).

### GSA Classroom

GSA Classroom is a gateway to slides, videos, and animations to learn GSA. In its first version, it is made of links to some online courses (both slides and animations) and a database to find online videos.

Our GSA animated lessons are seven lessons covering annotation databases, over-representation analysis (ORA), and functional class scoring (FCS), which include both theoretical knowledge and software usage.

**FIGURE 3:**
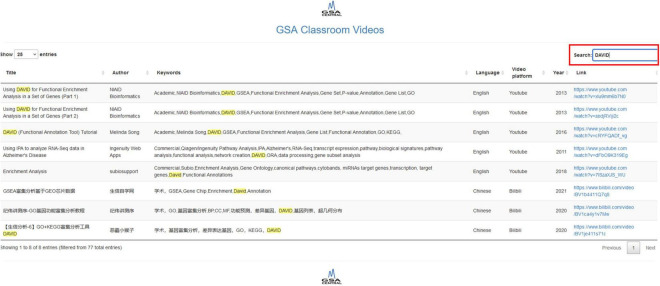
GSA Classroom screenshot. In this example, we searched for the keyword “DAVID” and found all online videos where it appears either in the title or in the keyword columns.

Our shiny app allows us to search by title, author, keywords, language, video platform, and year. It does feature a “Search” button that retrieves all the rows in the table whose information partially matches the query. Each column has an option that allows ordering in ascending or descending order (two small arrows next to the column name). The database includes both academic software and proprietary software, as well as English and Chinese videos. The first version of the video database contains 77 resources, 38 in English and 39 in Chinese, with 60 videos corresponding to academic works and 17 corresponding to commercial products. Figure 3 shows screenshots of GSA Classroom.

### GSARefDB

GSARefDB is the most comprehensive database of GSA methods and tools currently available. It is available as an excel file or as a shiny app, and contains information regarding the publication, website or software tool (if they exist), and an index of each paper’s popularity.

GSARefDB is made of seven main tables or menu options:

(i) “General methods and tools” contains information about general GSA methods, platforms, and software tools, usually applied to mRNA data.
(ii) “Reviews and benchmarks” includes information on reviews of the field and tool comparisons.
(iii) “Genomic GSA” contains information on methods and tools for GSA applied to genomic range data, such as ChIP-seq data, SNP data, and DNA methylation data.
(iv) “ncRNA GSA” includes methods and tools for GSA applied to ncRNA data, such as miRNA, lncRNA, and others.
(v) “MS-based GSA” includes methods and tools for GSA applied to proteomic, metabolomic, glucomic, and lipidomic data.
(vi) “Metagenomics GSA” includes methods and tools for GSA applied to metagenomic and metatranscriptomic data.
(vii) “Integromics GSA” includes methods and tools for GSA of experiments involving multiple *omics* datasets.

The app also includes a few other menu options, such as a “FAQ” tab and an “Analysis” tab (which offers summary plots of the entire database).

**FIGURE 4:**
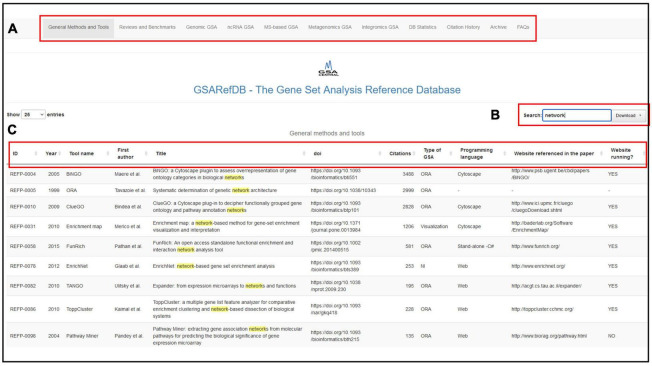
GSARefDB screenshot. **(A)** Main Menu: The user can choose between the database of general methods and tools, the database of reviews and benchmarks, the specialized databases (genomic GSA, nc-RNA GSA, MS-based GSA, meta-omics GSA, and integromics GSA), general GSARefDB statistics, the archive, and FAQs. **(B)** The search box: Here, we search for papers with the keyword “network”. **(C)** The columns with all the information we can obtain from each paper: ID, publication year, tool name, first author, paper title, DOI, number of citations, type of GSA, programming language, and website info.

Each of the tables has columns with relevant information, including method or tool name, reference (title, author, year, DOI), popularity (citation count), type of GSA, programming language, and website information. In the shiny app, each of the seven tables has a “Search” and a “Download” button to explore that table only. The search retrieves all the rows whose information partially matches the query. Each column in each table has an option for ordering that column in ascending or descending order (two small arrows next to the column name). For example, to see the popularity ranking of GSA tools, we can order the “Citations” column in descending order. A statistical summary, including the number of collected papers and the amount of collective citations by such papers, can be found in Table 2. Figure 4 shows screenshots of GSARefDB.

**TABLE 2:** Statistics of GSARefDB v.2.0.

	General methods and tools	Reviews and benchmarks	Genomic GSA	ncRNA GSA	MS-based GSA	Meta-omics GSA	Integromics GSA	Total
Number of papers	386	85	65	33	29	23	20	641
Number of citations	131,332	21,727	9,657	4,329	8,656	17,985	1,065	194,751

### GSA BenchmarKING

“GSA BenchmarKING” is a collection of tools to benchmark groups of GSA methods. Benchmarking tools include jupyter notebooks (with full workflows for benchmarking GSA methods) and shiny apps that allow benchmarking with the click of a few buttons. In the first version, we include two Jupyter notebooks (for “single-sample GSA” and “genomic-region GSA,” respectively) and two shiny apps (also for single-sample GSA and genomic-region GSA). Here we will explain the single-sample tools (Jupyter notebook and shiny app).

The Jupyter notebook compares five single-sample GSA methods [PLAGE (25), ZSCORE (26), SSGSEA (27), GSVA (20), and GRAPE (28)] plus an ensemble of all five. The workflow is designed to predict the pathways for four respiratory diseases that we know beforehand (non-small cell lung cancer, chronic obstructive pulmonary disease, asthma, and tuberculosis). In the beginning, datasets are downloaded and formatted to be read by the different methods. Then, each of the datasets is used as input of each of the methods to produce one predicted pathway ranking per dataset per method; in addition, a combination of their *p*-values is used as a sixth method to produce additional rankings. After that, each ranking is compared to the “target pathways” (the reported pathways for the four diseases) and precision, sensitivity, and specificity, are computed as specified before. Finally, box plots are built to compare the performance of each of the methods and each performance metric. With a minimum knowledge of R programming, Jupyter notebooks (or Rstudio notebooks) allow any part of the workflow to be modified; for example, using different datasets/gold standards or adding new GSA methods to the comparison.

The shiny app (“ss-shiny”) compares the same methods but gives more flexibility to a non-experienced user: The app allows the user to upload disease-related RNA expression datasets, either from a sample file for illustration purposes, the gold standard of disease-related RNA expression datasets introduced by Tarca et al. (29), or the user’s own dataset. The app also allows the user to determine its own disease/target pathways, either from a sample file or its own pathway file. The user can choose any number of methods among the five methods provided, as well as any method for *p*-value combination. Finally, the user can also choose the performance metrics (precision, sensitivity, specificity) to be evaluated. The app offers three tabs to, respectively, preview the input datasets, preview the results, and download the results, together with a menu containing an example of a benchmark study and a help menu.

**FIGURE 5:**
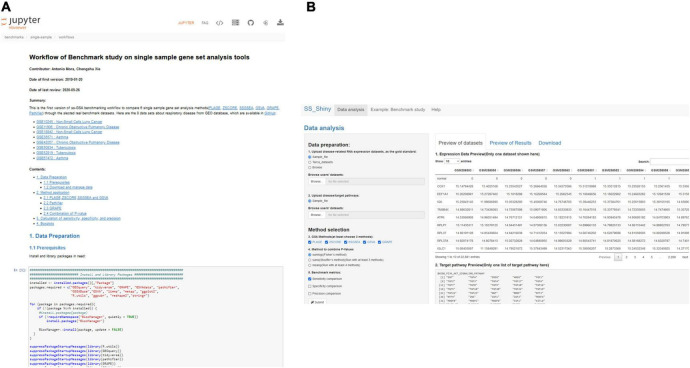
GSA BenchmarKING screenshot. **(A)** A jupyter notebook with an R workflow for the benchmark of six different single-sample GSA tools using datasets related to respiratory disease. **(B)** A shiny app to benchmark five different single-sample GSA tools using different gold standards, target pathways, and benchmark metrics.

Benchmarks and performance evaluation are not an easy task, and there is abundant discussion regarding which should be the parameters to compare and the procedures to follow (1), but “GSA BenchmarKING” makes such procedures transparent and allows the user to change every aspect they disagree with, which nowadays is not easy due to the closed ways in which benchmarks are practiced. We expect “GSA BenchmarKING” can become a place, or at least an inspiration, for more advanced users to develop their own open benchmarks or to validate their own GSA methods. Figure 5 shows some screenshots of GSA BenchmarKING.

### GSA Blog

We introduced “GSA Blog” as a space to share current and useful information related to the GSA field. Most specifically, at the GSA Blog, we will share posts including discussions on both old and new GSA methods and tools, as well as useful code.

Our initial posts included discussions on gene set annotation databases, how to understand and handle *p*-values, time-course GSA tools, and single-cell GSA tools.

In the future, we expect “GSA Blog” to become a space open to the GSA community.

## Discussion

We have built an education and research resource useful for GSA beginners, average users, and tool creators alike. Beginners have access to “GSA Classroom,” where they can watch lessons, conferences and other types of videos for different types of GSA or even for specific academic or commercial software. Average users can use “Galaxy-GSA,” a Galaxy instance with multiple GSA tools, including tools to download reference databases, generic popular GSA tools, and tools tailored to analyze single-sample designs, genomic regions, DNA methylation, integrative *omics* datasets, and others. Expert users, that is, creators of GSA methods and tools, may find that “GSA BenchmarKING” is a useful resource to test their methods compared to already tested methods. Both GSA average users and tool creators will find useful to visit “GSARefDB,” a database that allows the user to explore all available papers, finding all available reviews or benchmarks, finding all tools written for a specific type of analysis or programming software, discovering the popularity of methods and tools, and other information that will help the user make better decisions regarding method and tool selection. Finally, all three types of users might find it interesting to access “GSA Blog,” a space to post comments on current GSA software and current discussions of interest.

We have designed this platform with the purpose of covering all different types of resources that users of a given scientific field might be looking for, such as (i) introductory learning, (ii) state-of-the-art updates, (iii) method/tool selection guidelines and insights, (iv) easy-to-use tools, (v) tool integration strategies (Galaxy environment), (vi) tool design examples, and (vii) method/tool validation/benchmarking tools. Therefore, we introduce “GSA Central” as an example of a “thematic platform” containing all the resources that researchers might need in a given field.

“GSA Central” tools are relatively easy to update and maintain. Therefore, we plan to keep adding more methods and workflows in the near future.

In a previous paper, we suggested that “GSARefDB” and “GSA BenchmarKING” can be seen as the instruments of a methodology to permanently follow up the popularity and the performance status of all the tools in any bioinformatics sub-field (1). Going one step beyond, we believe that a platform like “GSA Central” is more than a website and can be considered as the main instrument of a novel approach to systematically and comprehensively contribute to the development of any scientific discipline.

## Software availability

“GSA Central” can be found at: https://gsa-central.github.io/.

“Galaxy-GSA” can be found at https://gsa-central.github.io/galaxy.html (general page), http://www.moralab.science:8080/ (web platform), https://github.com/gsa-central/galaxy-gsa/tree/main/docker (Docker image), and https://zenodo.org/record/5091267#.Yl5wXNNByUk (Virtual machine).

“GSA Classroom” can be found at https://gsa-central.github.io/education.html.

“GSARefDB” can be found at https://gsa-central.github.io/gsarefdb.html.

“GSA BenchmarKING” can be found at https://gsa-central.github.io/benchmarKING.html.

“GSA Blog” can be found at https://gsa-blog.github.io/index.html.

“Galaxy-GSA” has also been included as part of the “Galaxy community hub” (https://galaxyproject.org/use/galaxy-gsa/).

“GSA Central” contains open software under a Creative Commons license.

## Data availability statement

The original contributions presented in this study are publicly available. This data can be found here: https://gsa-central.github.io/.

## Author contributions

XH and AM built the GSA Central’s website. Galaxy-GSA was designed by AM and programmed by XH (including the individual tools, website, Docker image, and virtual machine). SM provided Galaxy and server technical support. GSA classroom was conceived by AM and built by XL and ZX based on code written by CX. GSA animated lessons were written by AM and programmed by XL. GSARefDB was conceived and built by AM, and its shiny app was written by CX. GSA BenchmarKING was conceived by AM and built by CX and SJ. GSA Blog was created by AM and its website was assembled by XH. AM conceived and supervised the project and wrote the manuscript. All authors contributed to the article and approved the submitted version.

## Footnotes

1
https://gsa-central.github.io/

2
https://usegalaxy.org

3
https://galaxyproject.org/use/

4
https://galaxyproject.org/toolshed/

5
https://planemo.readthedocs.io/en/latest/

6
https://github.com/gsa-central/galaxy-gsa/blob/main/tutorials/using-galaxy-gsa.md

7
https://github.com/mora-lab/installing/tree/main/galaxy

8
https://github.com/mora-lab/installing/tree/main/planemo

9
https://www.vyond.com/

10
https://www.adobe.com/products/aftereffects.html

11
https://github.com/gsa-central/gsaclassroom/blob/master/data/videos.xlsx

12
https://gsa-central.github.io/benchmarKING.html

13
https://gsa-blog.github.io

14
https://toolshed.g2.bx.psu.edu/

15
https://github.com/gsa-central/galaxy-gsa/tree/main/docker

16
https://zenodo.org/record/5091267#.Yl5wXNNByUk

17
https://gsa-central.github.io/galaxy.html
